# Supplementary figures and images for: Subject validation of reusable N95 stop-gap filtering facepiece respirators in COVID-19 pandemic
Source: PLoS One. 2020 Nov 13;15(11):e0242304. doi: 10.1371/journal.pone.0242304 (PMC7665821; doi:10.1371/journal.pone.0242304)

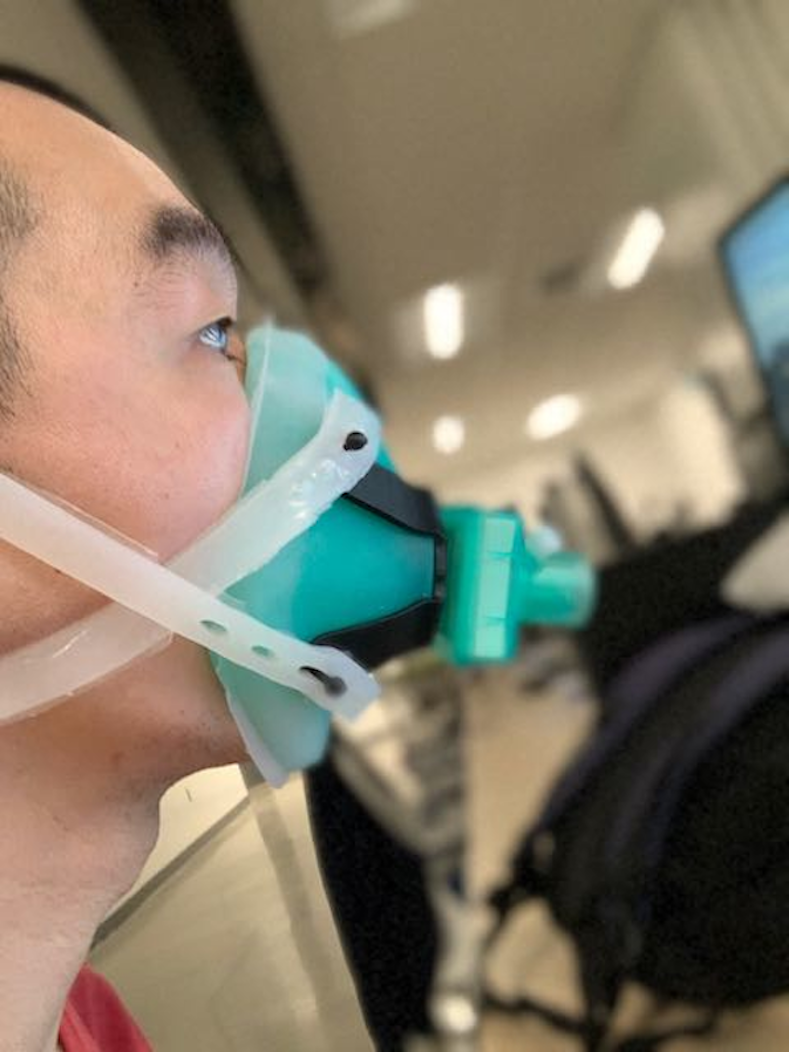

Supplement: S1 Fig — (TIF) [file pone.0242304.s001.tif]
